# *Streptococcus anginosus* extracellular vesicles deliver protein to promote gastric cancer progression

**DOI:** 10.3389/fcimb.2026.1917293

**Published:** 2026-09-30

**Authors:** Feng Li, Xiling Chen, Hao Huang, Xiaowei Sun, Maoyuan Wang, Haoyuan Xie, Jian Xie, Yunrui Dong, Weize Li, Meiyan Wu, Hongying Fan, Yang Bai

**Affiliations:** 1 Guangdong Provincial Key Laboratory of Gastroenterology, Department of Gastroenterology, Institute of Gastroenterology of Guangdong Province, Nanfang Hospital, Southern Medical University, Guangzhou, China; 2 Department of Microbiology, Guangdong Provincial Key Laboratory of Tropical Disease Research, School of Public Health, Southern Medical University, Guangzhou, China; 3 Department of Gastroenterology, Sun Yat-Sen Memorial Hospital, Sun Yat-Sen University, Guangzhou, China; 4 Department of Gastroenterology, Baiyun District People’s Hospital of Guangzhou, Guangzhou, Guangdong, China

**Keywords:** bacteria-host communication, extracellular vesicles, gastric cancer, Streptococcus, *Streptococcus anginosus*

## Abstract

**Background:**

Gastric cancer (GC) is a leading cause of cancer-related mortality worldwide. Although *Helicobacter pylori* (Hp) is considered a major etiological factor, only 3% of Hp-infected individuals develop GC, implying additional microbial drivers. *Streptococcus anginosus* (SA) exhibits independent pro-carcinogenic properties, but its mechanism in regulating GC malignant phenotypes remains elusive. This study investigated the roles and molecular mechanisms of SA-derived extracellular vesicles (SA-EVs) in GC progression.

**Methods:**

SA-EVs were isolated and characterized, followed by *in vitro* and *in vivo* functional assays, transcriptomic analysis and rescue experiments to explore their pro-tumor effects and key components.

**Results:**

SA-EVs were efficiently internalized by GC cells, which markedly promoted GC cell proliferation, migration and invasion *in vitro*, and accelerated tumor growth *in vivo*. Mechanistically, the protein cargos of SA-EVs specifically triggered the activation of the JAK-STAT signaling pathway, which further upregulated the expression of genes associated with cell proliferation and epithelial-mesenchymal transition (EMT). Moreover, pharmacological inhibition experiments verified that removal of SA-EV protein components or pharmacological blockade of JAK-STAT signaling effectively eliminate the pro-tumor effects of SA-EVs.

**Conclusions:**

Streptococcus serves as a core signature genus in Hp-independent GC. SA drives GC malignancy by delivering functional proteins via EVs to activate JAK-STAT signaling. This study reveals a novel bacteria-host interaction mechanism mediated by SA-EVs, providing a potential target for GC intervention.

## Graphical Abstract

**Figure:**
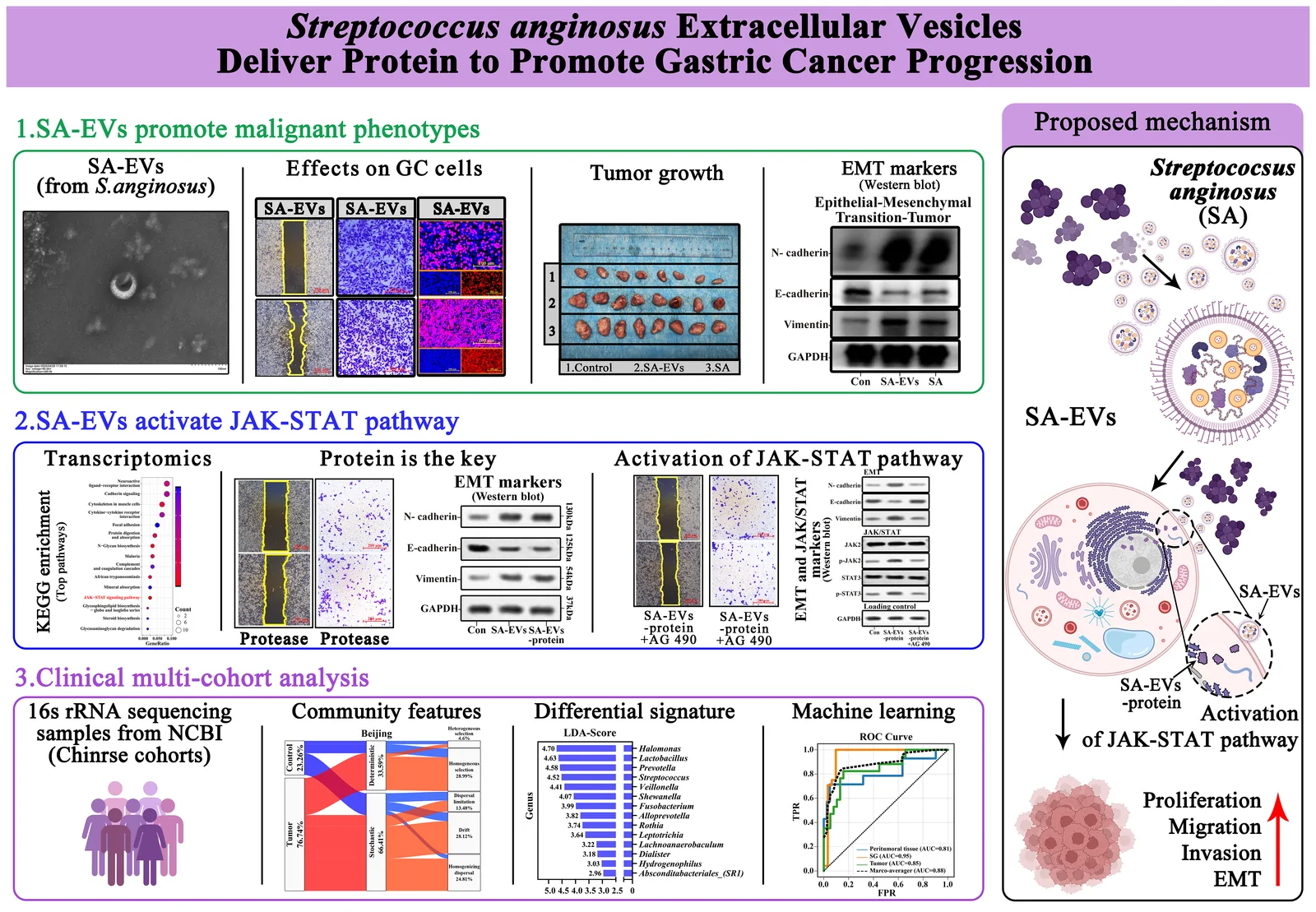
Created with Biorender.com.

## 1 Introduction

Gastric cancer (GC) ranks as the fifth most common cancer globally, accounting for 4.9% of all new cancer cases and 6.8% of cancer-related deaths worldwide, with over one million new cases annually, thereby posing a major public health burden (Yang et al., 2020; Bray et al., 2024). *Helicobacter pylori* (Hp) is classified as a Group 1 carcinogen for GC by the World Health Organization (Polk and Peek, 2010). However, in the real world, although approximately 50% of the global population is infected with Hp, only about 3% of infected individuals develop GC (Li et al., 2015). This epidemiological gap suggests that while Hp infection is a key driving factor for the occurrence and development of GC, it is not the sole prerequisite (Kumar et al., 2020; Liou et al., 2020; Bakhti and Latifi-Navid, 2021). Recent studies have revealed that as Correa’s cascade progresses toward the GC stage, the gastric microecology exhibits dynamic succession. Hp, which is originally dominant, undergoes paradoxical depletion (Ferreira et al., 2018; He et al., 2022), whereas opportunistic pathogens such as the genus Streptococcus and Prevotella emerge as core microbiota to reshape the gastric microbial structure in GC (Abate et al., 2022), indicating that GC possesses a unique microecological profile.

Among the GC-specific microbiota, *Streptococcus anginosus* (SA) has garnered significant attention due to its notable independent carcinogenic capacity (Fu et al., 2024). As a Gram-positive facultative anaerobic coccus, SA normally colonizes the oral cavity, gastrointestinal tract, and genitourinary tract, acting as an important opportunistic pathogen (Pilarczyk-Zurek et al., 2025). Recent studies have confirmed that SA was significantly enriched in the gastric mucosa of GC patients and droves the GC process through various mechanisms, including inducing chronic inflammation, disrupting the epithelial barrier, promoting abnormal cell proliferation, and inhibiting CD8^+^ T cell infiltration (Fu et al., 2024; Yuan et al., 2024). Zhou et al. further revealed that SA mediated methionine biosynthesis through its key metabolic gene metE, thus promoting GC progression (Zhou et al., 2026). Another study indicated that succinate, a metabolite derived from SA, can activate the PI3K/AKT signaling pathway via the SUCNR1/ABRAXAS1 axis to promote gastric tumorigenesis (Mehanny et al., 2020; Liao et al., 2026). These findings indicate that SA exerts tumor-promoting effects through multiple effector molecules.

Bacterial extracellular vesicles (EVs), secreted by bacteria as spherical lipid bilayer structures with a diameter of approximately 20–250 nm, widely carry active molecules such as nucleic acids, proteins, and lipids (Ahmadi Badi et al., 2019). They not only mediate inter-bacterial communication but also directly regulate immune responses and inflammatory reactions by delivering their contents to host cells, becoming a key focal point for elucidating bacteria-host interactions. Gong et al. systematically investigated the pathogenic role of SA-EVs in gastritis, providing critical mechanistic insights into SA-EV-mediated non-neoplastic gastric mucosal inflammation (Gong et al., 2026). Xie et al. further elucidated that SA-EVs delivered the bacterial chromatin remodeler saSNF2 into the host cell nucleus, where it cooperated with the host transcription factor TEAD1 and the BAF complex to upregulate the expression of the palmitoyltransferase ZDHHC11, which in turn established an immunosuppressive microenvironment through PD-L1 palmitoylation and promoted GC progression (Xie et al., 2025). This work provides a refined molecule mechanistic explanation for SA-EV-mediated immune evasion in GC from the perspective of epigenetic regulation.

Despite these important advances, several key scientific questions remain unanswered. First, the overall regulatory effects of SA-EVs on the malignant phenotypes of GC and the associated signaling pathway networks remain poorly characterized while existing studies primarily focus on the single effector molecule saSNF2, and whether other key active molecules in SA-EVs contribute to the carcinogenic process remains unclear. Second, the issue of microbial heterogeneity across studies due to differences in sampling sites and population backgrounds has not been systematically evaluated. The niche position of the genus Streptococcus in the gastric tumor microecology and the strength of its clinical association still lack multi-cohort consistent validation.

It is worth noting that while current gastric microbiota research has made considerable progress utilizing metagenomics and 16S sequencing technologies, the accumulation of data is far less than that of the gut microbiota, and there are significant differences in study designs and sample collection methods. Existing sample types for GC microbiome research encompass the gastric antrum mucosa, gastric corpus mucosa, gastric juice, and even different lesion areas (Maubach et al., 2025). Differences in sampling sites regarding acidity and exposure to bile reflux may lead to inconsistencies in microbiota composition and abundance structures. Furthermore, variations in dietary patterns among populations in different regions further exacerbate cross-study microbial heterogeneity. Therefore, although multiple cohorts have reported enrichment trends of potential pathogenic bacteria in gastric precancerous lesions or advanced stages, issues such as inconsistent conclusions across studies and insufficient reproducibility are prevalent (Wizenty and Sigal, 2025; Chang et al., 2026).

Based on this, the present study systematically investigates the role of SA-EVs in GC and their active molecules. First, we integrate 16S rRNA sequencing data from multiple Chinese cohorts of gastric antrum mucosa to systematically characterize the structural shifts of the gastric microbiota from healthy states to GC from a macroecological perspective, and employ the random forest and SHAP framework to clarify the niche role of the genus Streptococcus in GC progression, providing population-level ecological context for the study of SA (it should be noted that 16S rRNA sequencing can only reliably resolve taxa at the genus level and cannot distinguish specific species within the genus Streptococcus, detailed analyses were presented in the **Supplementary Materials**). Building on this foundation, we isolate and characterize SA-EVs, systematically investigate their regulatory effects on the malignant phenotypes of GC and the key signaling pathway networks involved, and employ a component isolation and functional screening strategy to identify key active molecules in SA-EVs that exert pro-carcinogenic effects. This study seeks to systematically characterize the biological effects of SA-EVs at the holistic vesicle level and conduct unbiased screening for their active components.

## 2 Methods

### 2.1 Bacterial strains and cell lines

The SA strain used in this study was ATCC 33397, obtained from the Department of Microbiology, School of Public Health, Southern Medical University. In an anaerobic environment, SA was cultured in MRS broth medium (Cat: 027315, HKM) within an incubator at 37 °C with 5% CO_2_. Human GC cell lines AGS and MKN-45 were obtained from the Experimental Center of Nanfang Hospital, Southern Medical University. Both cell lines were cultured in RPMI 1640 medium (Cat: C11875500BT, Gibco) supplemented with 10% FCS (Cat: A5256701, Thermo Fisher Scientific) and 1% penicillin-streptomycin solution (Cat: 15070063, Thermo Fisher Scientific) in an incubator at 37 °C with 5% CO_2_.

### 2.2 Extraction and quantification of SA-EVs

SA was cultured in MRS broth medium to the stationary phase, and the culture was then centrifuged at 10,000 g for 30 min at 4°C to remove bacterial cells. The resulting supernatant was filtered through a 0.22μm filter to remove residual bacterial fragments and other large particulate matter. The filtrate was further centrifuged at 100,000 g for 60 min at 4°C. The supernatant was carefully discarded, and the SA-EVs at the bottom of the centrifuge tube were resuspended in phosphate-buffered saline (PBS, Cat: PBS, ACMEC). BCA Protein Assay Kit (Cat: 23225, Thermo Fisher Scientific) was used to quantify the protein concentration of the obtained SA-EVs suspension, utilizing bovine serum albumin (BSA, Cat: A104912, Aladdin) as the standard. The standards and the PBS-diluted SA-EVs samples were sequentially added to a 96-well plate along with the BCA working solution. After mixing, the plate was incubated in the dark at 37°C for 30 min. The absorbance was measured at a wavelength of 562 nm, and the protein concentration of SA-EVs was calculated based on the BSA standard curve.

The remaining detailed experimental methods can be found in the **Supplementary Materials**.

## 3 Results

### 3.1 Isolation and characterization of SA-EVs

To investigate the role of SA in promoting GC, SA EVs were extracted and characterized. Transmission electron microscopy revealed that purified SA-EVs exhibited a typical cup shaped or concave morphology (**Figure 1A**). NTA further showed that their size distribution predominantly ranged from 100 to 200 nm (**Figure 1B**). Western blot confirmed the enrichment of lipoteichoic acid (LTA), a marker of Gram positive bacterial extracellular vesicles, in SA-EVs (**Figure 1C**). Collectively, these results confirmed the integrity, size distribution, and characteristics of SA-EVs.

**Figure 1:**
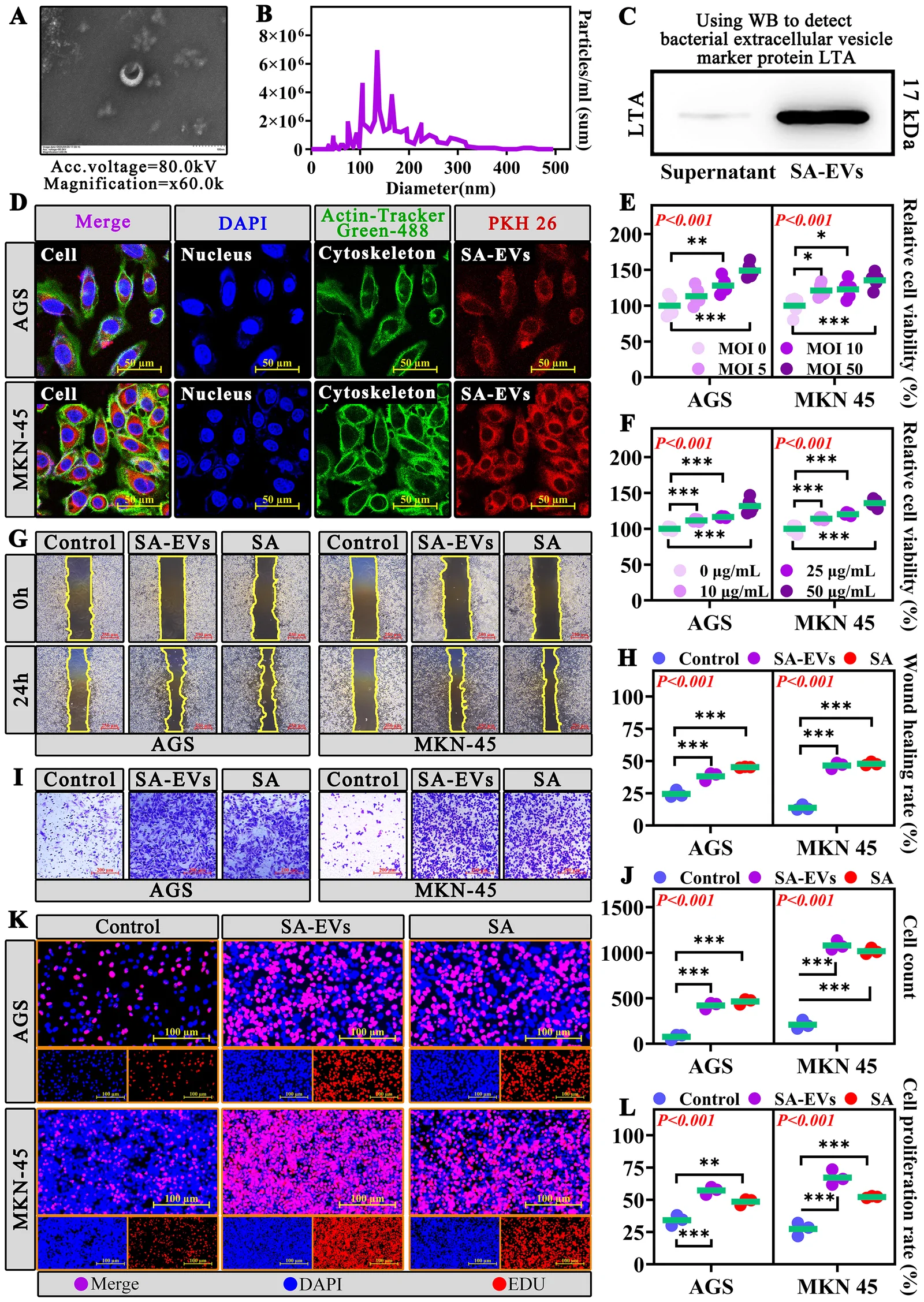
Isolation and characterization of SA-EVs and the effects on malignant phenotypes of GC cells. **(A)** Transmission electron microscopy image of purified SA-EVs (scale bar: 100 nm). **(B)** Particle size distribution curve of SA-EVs determined by NTA. **(C)** Western blot detection of LTA, the marker of Gram-positive bacterial extracellular vesicles. **(D)** Confocal microscopy images showing efficient internalization of PKH26-labeled SA-EVs (red) by human GC cells (AGS and MKN-45). Actin cytoskeleton and nuclei were stained with phalloidin (green) and DAPI (blue), respectively (scale bar: 50 μm). **(E, F)** Cell viability of AGS and MKN-45 cells treated with different concentrations of SA or SA-EVs for 24 h, assessed by CCK-8 assay. **(G, H)** Wound healing assay and quantitative analysis showing that SA-EV treatment significantly enhances the migration ability of AGS and MKN-45 cells (scale bar: 250 μm). **(I, J)** Transwell invasion assay and quantitative analysis showing that SA-EV treatment significantly enhances the invasion ability of AGS and MKN-45 cells (scale bar: 200 μm). **(K, L)** EdU assay and quantitative analysis showing that SA-EV treatment significantly increases the proliferative activity of AGS and MKN-45 cells (scale bar: 100 μm). Data are presented as mean ± SD, n = 3. ns *P* > 0.05, *P ≤ 0.05, **P ≤ 0.01, ***P ≤ 0.001. Created with Biorender.com.

### 3.2 SA-EVs are efficiently internalized by GC cells and promote cell viability in a dose-dependent manner

To characterize the interaction between SA-EVs and GC cells, PKH26-labeled SA-EVs were co-cultured with AGS and MKN-45 cells. Confocal microscopy revealed that SA-EVs were efficiently internalized and predominantly localized in the cytoplasmic region (**Figure 1D**). The CCK-8 assay was used to evaluate the effects of varying concentrations of SA and SA-EVs on GC cell viability after 24 h of treatment. The results showed that the viability of both GC cell lines increased in a gradient manner with increasing concentrations of SA and SA-EVs (**Figures 1E, F**), peaking at an MOI of 50 for SA and an SA-EV concentration of 50 μg/mL. These findings clarify the mode of SA-EV entry into GC cells and establish the optimal intervention concentration for their biological effects.

### 3.3 SA-EVs enhance the migration, invasion, and proliferation of GC cells

To further elucidate the impact of SA-EVs on the malignant phenotype of GC, AGS and MKN-45 cells were co-cultured with SA-EVs, followed by wound healing, Transwell invasion, and EdU incorporation assays to assess changes in cell migration, invasion, and proliferation, respectively. The results showed that SA-EV treatment significantly promoted the migration and invasion abilities of both GC cell lines (**Figures 1G–J**), and concomitantly increased the proportion of EdU-positive cells, indicating enhanced proliferative capacity (**Figures 1K, L**). Collectively, these functional assays demonstrate that SA-EVs enhance the migration, invasion, and proliferation of GC cells *in vitro*, providing initial evidence for their driving role in GC malignant progression.

### 3.4 SA-EVs promote GC growth and induce epithelial-mesenchymal transition *in vivo*

To further validate the pro-tumorigenic effects of SA-EVs in the physiological microenvironment, subcutaneous mouse tumor model of GC was established, and mice were treated with intratumoral injection of SA-EVs or SA bacterial suspension (**Figure 2A**). The results showed that, compared with the control group, both the SA-EVs and SA groups significantly promoted tumor growth, as evidenced by markedly increased tumor volume and weight (**Figures 2B, C**), indicating that SA-EVs exert tumor-promoting effects *in vivo* comparable to those of live bacteria. Western blot and immunohistochemical analyses revealed that SA-EVs and SA treatment significantly upregulated the expression levels of the proliferation-associated markers PCNA and Ki-67 in tumor tissues (**Figures 2D–F**), further confirming their pro-proliferative effects. Concurrently, both SA-EVs and SA treatment induced a typical switch in EMT-related markers. The mesenchymal markers N-cadherin and Vimentin were significantly elevated, whereas the epithelial marker E-cadherin was markedly downregulated (**Figures 2G–I**). Collectively, these findings demonstrate that SA-EVs not only directly promote GC cell proliferation *in vivo* but also drive malignant progression by inducing EMT, thereby altering the invasive phenotype of tumor cells.

**Figure 2:**
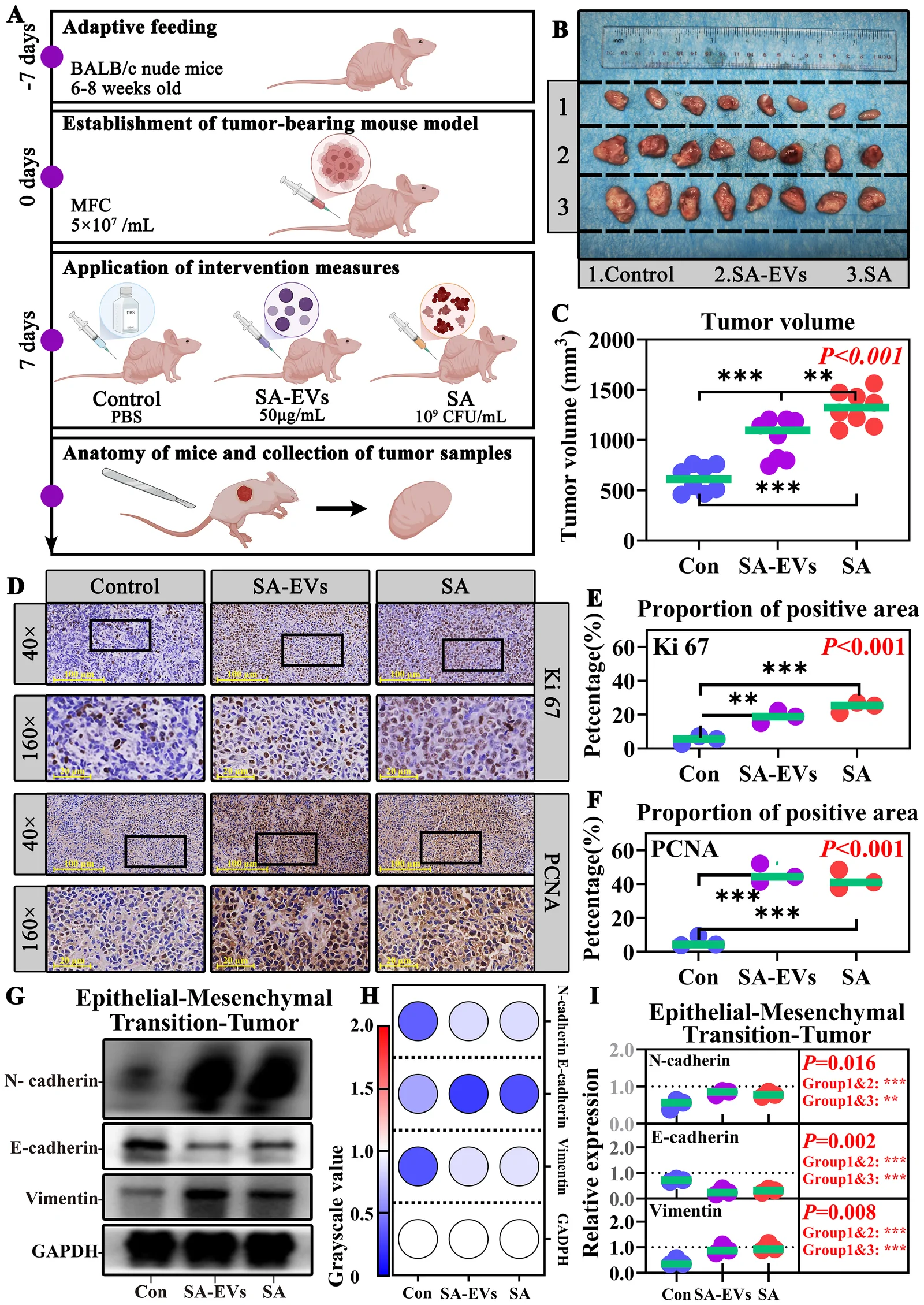
SA-EVs promote tumor growth, proliferation, and EMT in a tumor-bearing mice model. **(A)** Schematic diagram of the animal experiment. (2B-2C) Representative tumor images **(B)** and tumor volume **(C)** of control, SA-EVs-treated, and SA-treated groups (n=8). **(D–F)** Immunohistochemical staining **(D)** and quantitative analysis **(E, F)** of proliferation markers PCNA and Ki-67 in tumor tissues. **(G–I)** Western blot analysis **(G)** and quantitative analysis **(H, I)** of EMT-related markers (N-cadherin, Vimentin, and E-cadherin) in tumor tissues. Data are presented as mean ± SD, n = 3. ns *P* > 0.05, *P ≤ 0.05, **P ≤ 0.01, ***P ≤ 0.001. Created with Biorender.com.

### 3.5 Transcriptomic analysis reveals that SA-EVs promote GC progression via the JAK-STAT pathway

To elucidate the molecular mechanism by which SA-EVs drive GC malignant progression, transcriptomic sequencing was performed on tumor tissues from control and SA-EV-treated mice. Differential expression analysis identified a total of 557 differentially expressed genes (DEGs), of which 163 were significantly upregulated and 394 downregulated in the SA-EV group (**Figures 3A, B**).

**Figure 3:**
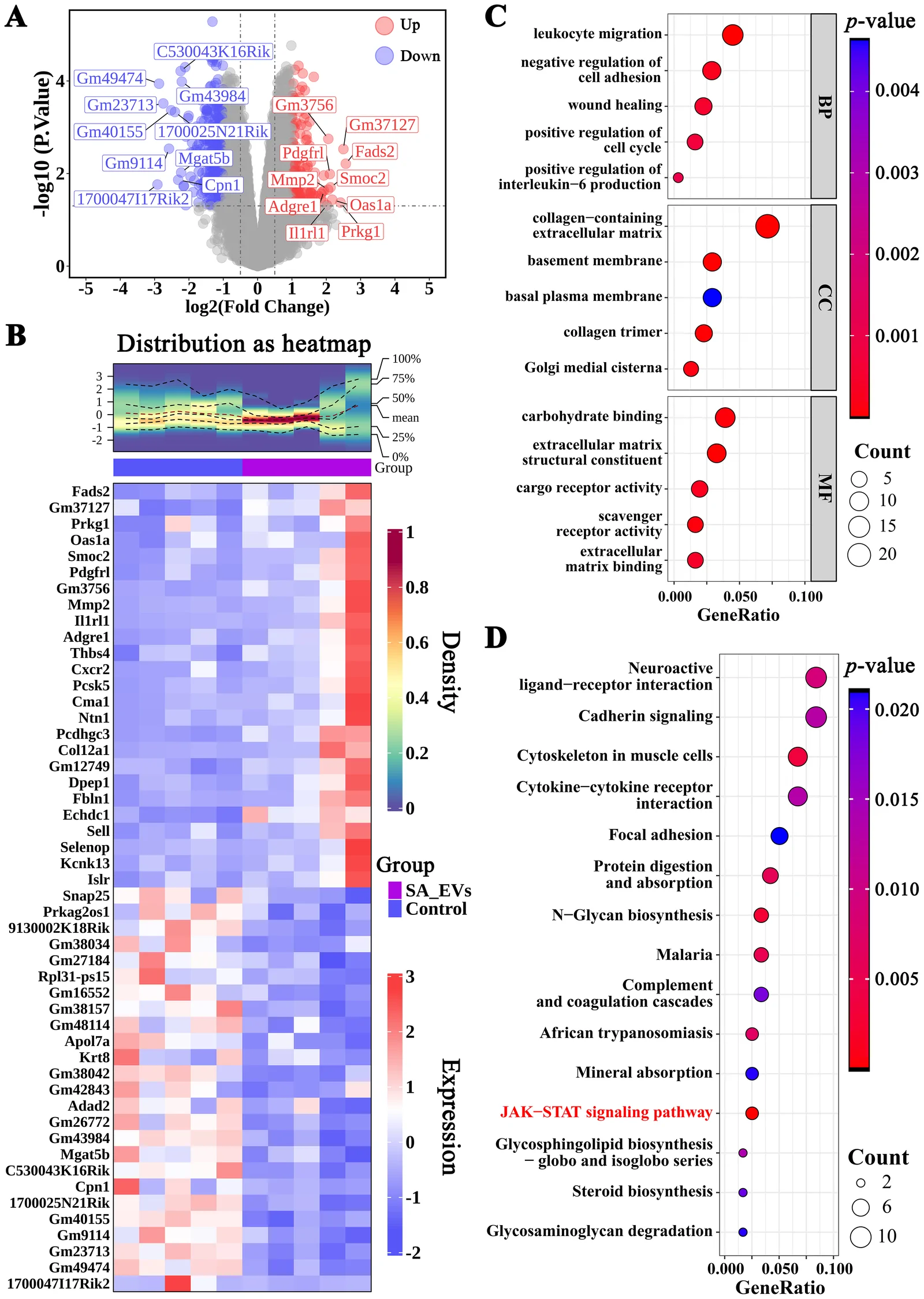
Transcriptomic analysis reveals that SA-EVs promote GC progression by activating the JAK-STAT signaling pathway. **(A)** Volcano plot showing differentially expressed genes in tumor tissues between the SA-EV-treated and control groups. Red and blue dots represent significantly upregulated (n = 163) and downregulated (n = 394) genes, respectively (|log_2_FC| ≥ 1, FDR<0.05). **(B)** Heatmap showing the expression patterns of 557 DEGs across samples from the two groups. **(C)** GO enrichment analysis of the DEGs. (3D) KEGG pathway enrichment analysis. Created with Biorender.com.

To further explore the biological functions of the DEGs, Gene Ontology (GO) and KEGG pathway enrichment analyses were conducted. GO analysis revealed that the DEGs were significantly enriched in terms related to “positive regulation of interleukin-6 production”, “positive regulation of cell cycle”, “collagen-containing extracellular matrix”, “negative regulation of cell adhesion”, and “leukocyte migration” (**Figure 3C**).

KEGG pathway enrichment analysis further demonstrated that the DEGs were significantly enriched in the JAK-STAT signaling pathway (**Figure 3D**). As a central hub connecting inflammatory signals to malignant tumor phenotypes, the enrichment of this pathway is highly consistent with GO terms such as “positive regulation of interleukin-6 production” and “positive regulation of cell cycle”.

Collectively, transcriptomic analysis reveals that SA-EVs may activate the JAK-STAT signaling pathway, thereby coordinately regulating multiple biological processes closely associated with tumor malignant progression, including inflammatory responses, cell cycle progression, and extracellular matrix remodeling, providing potential directions for subsequent mechanistic validation.

### 3.6 The protein cargo of SA-EVs is the key active component driving GC malignant progression

SA-EVs carry multiple components, including proteins, RNA, DNA, and lipids. To identify the key components mediating GC cell proliferation, migration, and invasion, we disrupted the intact vesicle structure of SA-EVs by repeated freeze-thaw cycles, followed by treatment with proteinase, RNase, DNase, or silica to specifically remove proteins, RNA, DNA, or lipids, respectively. The treated samples were then co-cultured with GC cells to evaluate their functional effects.

The results showed that freeze-thawed SA-EVs still significantly enhanced GC cell proliferation, migration, and invasion (**Figure 4**; **Supplementary Figure 1**), indicating that the pro-tumorigenic effect of SA-EVs does not depend on their intact vesicle structure, and the active components retain biological activity even after vesicle disruption. Further specific depletion of individual components revealed that proteinase-treated SA-EVs completely lost their ability to promote cell proliferation, migration, and invasion, whereas RNase, DNase, or lipid removal did not significantly affect their pro-tumorigenic effects (**Figures 4A–F**; **Supplementary Figures 1A–F**). Collectively, these results consistently demonstrate that the protein components are the core molecular basis underlying the pro-tumorigenic function of SA-EVs.

**Figure 4:**
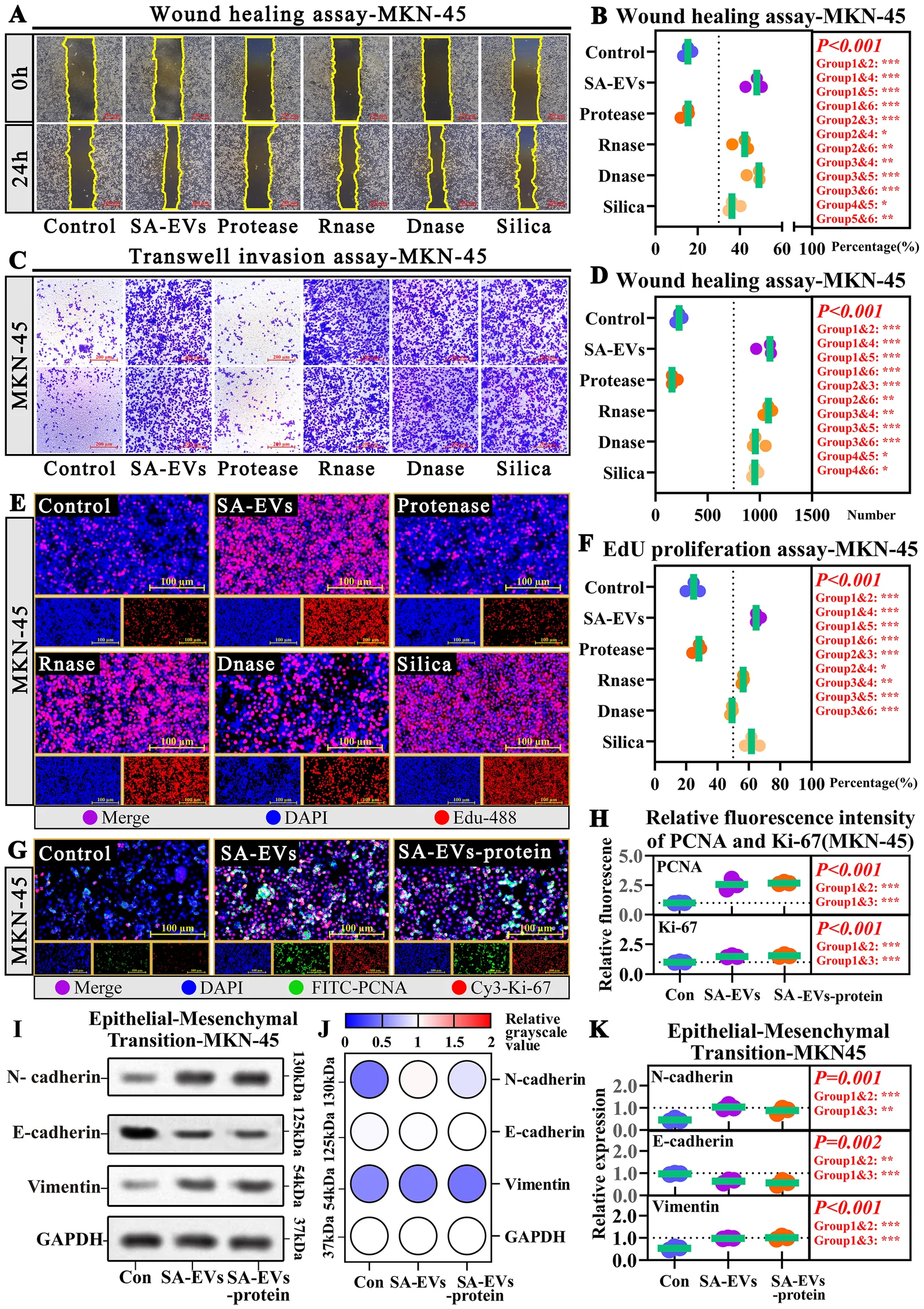
MKN-45 cells demonstrate that the protein cargo of SA-EVs is the key active component driving GC malignant progression. **(A, B)** Wound healing assay and quantitative analysis. SA-EVs were subjected to repeated freeze-thaw cycles to disrupt vesicle structure, followed by specific removal of proteins, RNA, DNA, or lipids using proteinase, RNase, DNase, or silica, respectively, and then co-cultured with MKN-45 cells. The protein cargo of SA-EVs significantly enhanced the migration ability of MKN-45 cells (scale bar: 250 μm). **(C, D)** Transwell invasion assay and quantitative analysis showing that the SA-EV protein cargo significantly enhanced the invasion ability of MKN-45 cells (scale bar: 200 μm). **(E, F)** EdU assay and quantitative analysis showing that the SA-EV protein cargo significantly enhanced the proliferative activity of MKN-45 cells (scale bar: 100 μm). **(G, H)** Immunofluorescence and quantitative analysis of proliferation markers PCNA and Ki-67 in MKN-45 cells treated with control, SA-EVs, or SA-EV protein cargo (scale bar: 100 μm). **(I–K)** Western blot analysis and quantitative analysis of EMT-related markers (N-cadherin, Vimentin, and E-cadherin) in MKN-45 cells treated with control, SA-EVs, or SA-EV protein cargo. Data are presented as mean ± SD, n = 3. ns *P* > 0.05, *P ≤ 0.05, **P ≤ 0.01, ***P ≤ 0.001. Created with Biorender.com.

Furthermore, after treating GC cells with the protein cargo of SA-EVs, we examined the expression changes of proliferation- and EMT-related markers by immunofluorescence and western blotting. The results showed that the SA-EV protein cargo significantly upregulated the expression of the proliferation markers PCNA and Ki-67 (**Figures 4G, H**; **Supplementary Figures 1G, H**), upregulated the mesenchymal markers N-cadherin and Vimentin, and downregulated the epithelial marker E-cadherin (**Figures 4I–K**; **Supplementary Figures 1I–K**). Together, these findings confirm that the protein cargo within SA-EVs is the key active component driving GC malignant progression.

### 3.7 The protein cargo of SA-EVs drives GC malignant progression by activating the JAK-STAT signaling pathway

Previous transcriptomic analysis suggested that SA-EVs may exert pro-tumorigenic effects through regulation of the JAK-STAT signaling pathway. To determine whether the protein cargo of SA-EVs mediates the malignant phenotype of GC via this pathway, we performed functional pharmacological inhibition experiments using AG490, a specific inhibitor of the JAK-STAT pathway (**Figure 5**; **Supplementary Figure 2**).

**Figure 5:**
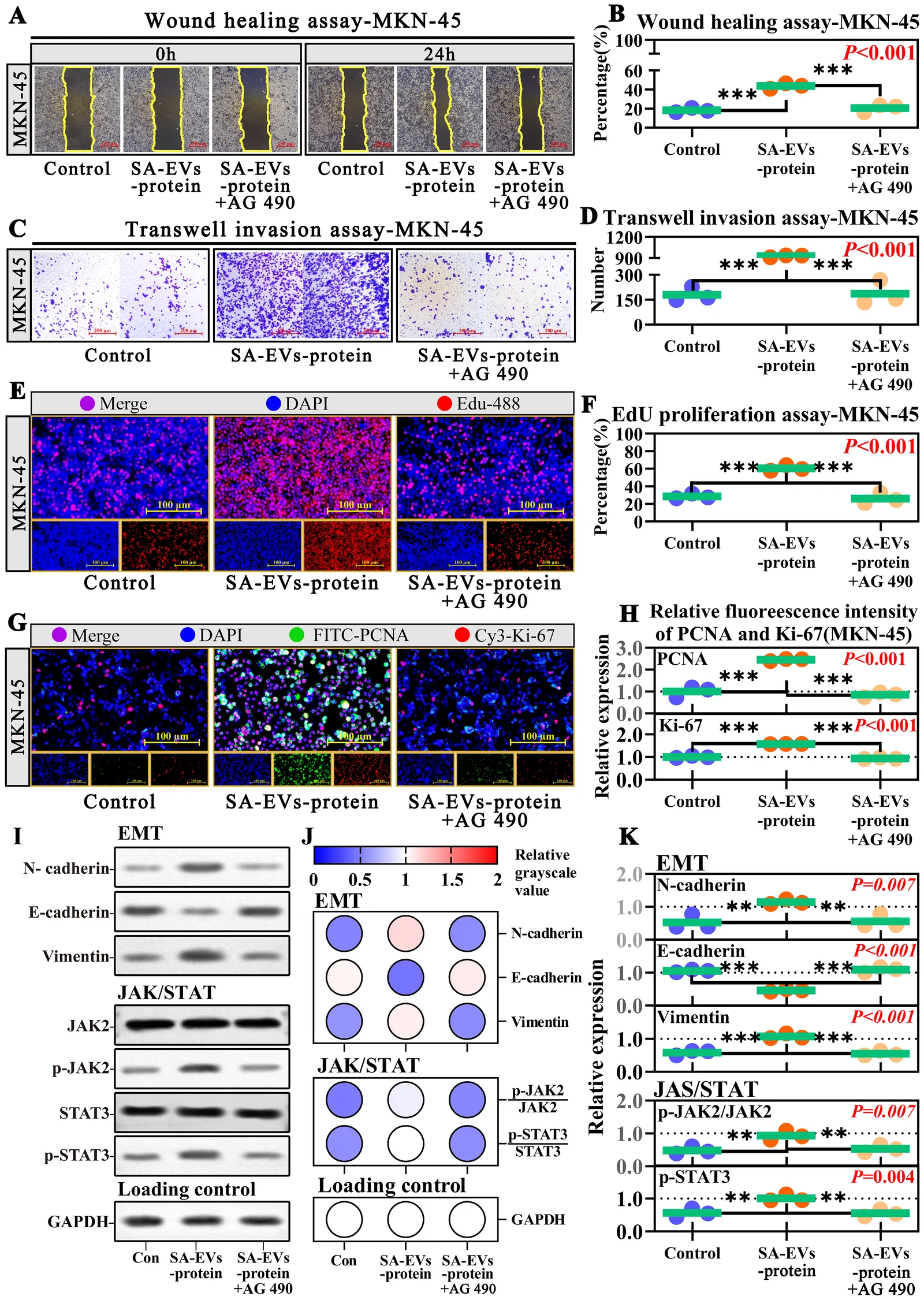
MKN-45 cells demonstrate that the protein cargo of SA-EVs drives GC malignant progression via the JAK-STAT pathway. **(A, B)** Wound healing assay and quantitative analysis. The SA-EV protein cargo significantly enhanced the migration ability of MKN-45 cells, and this effect was reversed by treatment with AG490, a JAK-STAT pathway inhibitor (scale bar: 250 μm). **(C, D)** Transwell invasion assay and quantitative analysis. The SA-EV protein cargo significantly enhanced the invasion ability of MKN-45 cells, and this effect was reversed by AG490 treatment (scale bar: 200 μm). **(E, F)** EdU assay and quantitative analysis showing that the SA-EV protein cargo significantly increased the proliferative activity of MKN-45 cells, and this effect was reversed by AG490 treatment (scale bar: 100 μm). **(G, H)** Immunofluorescence and quantitative analysis of proliferation markers PCNA and Ki-67 in MKN-45 cells treated with control, SA-EV protein cargo, or SA-EV protein cargo plus AG490 (scale bar: 100 μm). **(I–K)** Western blot analysis **(I)** and quantitative analysis **(J, K)** of EMT-related markers (N-cadherin, Vimentin, and E-cadherin) in MKN-45 cells treated with control, SA-EV protein cargo, or SA-EV protein cargo plus AG490. Data are presented as mean ± SD, n = 3. ns *P* > 0.05, *P ≤ 0.05, **P ≤ 0.01, ***P ≤ 0.001. Created with Biorender.com.

Compared with the control group, treatment with the SA-EV protein cargo significantly enhanced the proliferation, migration, and invasion abilities of GC cells. However, co-treatment with AG490 largely abolished these pro-tumorigenic effects (**Figures 5A–F**; **Supplementary Figures 2A–F**), indicating that intact activation of the JAK-STAT pathway is essential for the pro-tumorigenic function of the SA-EV protein cargo. Furthermore, the SA-EV protein cargo significantly upregulated the expression of the proliferation markers PCNA and Ki-67 and induced an EMT phenotype, as evidenced by increased expression of the mesenchymal markers N-cadherin and Vimentin and decreased expression of the epithelial marker E-cadherin. Notably, AG490 treatment effectively reversed these protein expression changes (**Figures 5G–K**; **Supplementary Figures 2G–K**). Collectively, these findings demonstrate that the protein cargo of SA-EVs promotes GC cell proliferation, migration, invasion, and EMT process by activating the JAK-STAT signaling pathway.

### 3.8 SA-EVs contain protein components with potential cytomodulatory and cell-interacting properties

To characterize the functional basis of SA-EVs, a proteomic analysis was performed. A total of 48 proteins were identified, including 10 proteins with counts>10 and 19 proteins with counts ranging from 2 to 10 (**Figure 6A**).

**Figure 6:**
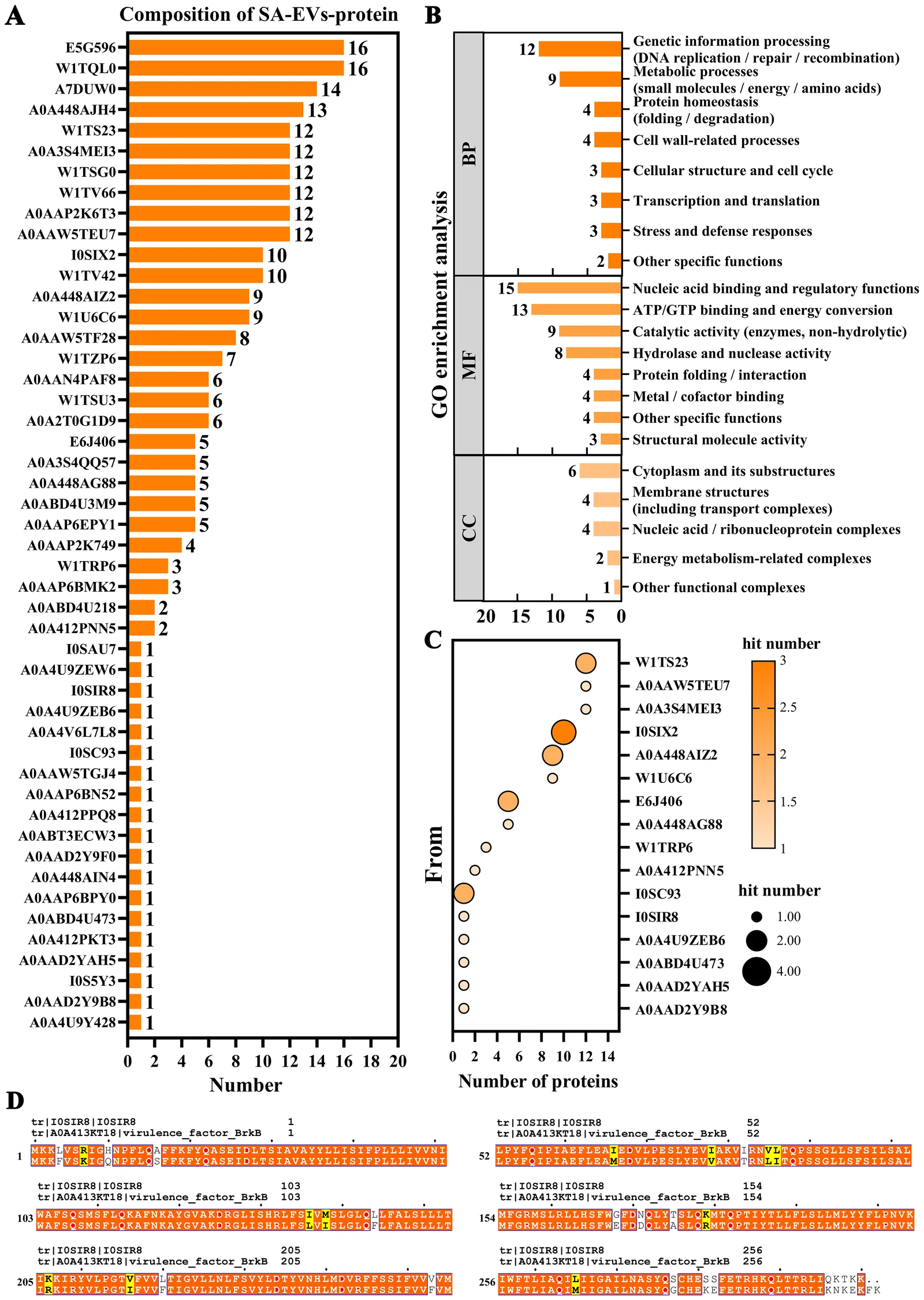
Proteomic identification of SA-EVs. **(A)** List of identified proteins in purified SA-EVs and their relative abundances determined by label-free quantitative proteomics (LC-MS/MS). **(B)** Gene Ontology (GO) enrichment analysis of the identified proteins in SA-EVs. GO annotation is divided into three standardized classification branches: biological process (BP) describes overall biological functions and physiological events executed by gene products within cells; molecular function (MF) refers to the intrinsic biochemical activities of individual proteins at the molecular level; cellular component (CC) defines the specific subcellular locations, protein complexes or extracellular structures where proteins reside. **(C)** Bubble plot showing the identified proteins that were mapped to injury-related GO terms. The x-axis represents the relative abundance of each protein. **(D)** Pfam domain architecture prediction of the potential injury-associated protein I0SIR8, revealing the presence of a virulence-associated BrkB domain. Created with Biorender.com.

GO analysis revealed that the identified proteins were predominantly enriched in pathways related to genetic information processing at the BP level. At the MF level, nucleic acid binding and regulatory functions were the most significantly enriched categories. However, annotations at the CC level were relatively limited and mainly associated with cytoplasmic structures (**Figure 6B**). To identify proteins potentially involved in tumor cell interaction and cytomodulatory effects, GO categories including stress and defense responses, nucleic acid binding and regulatory functions, hydrolase and nuclease activity, and membrane structures (including transport complexes) were defined as functionally relevant. Based on this strategy, 26 candidate proteins were initially selected. Subsequently, Pfam domain annotation was applied for further functional refinement. Proteins primarily associated with fundamental bacterial physiological processes, such as ribosomal proteins, RNA polymerase subunits, ATP synthase components, transcriptional regulators, and typical metabolic enzymes—were excluded. This resulted in a final set of 16 proteins with potential functional relevance (**Figure 6C**).

Notably, three relatively abundant proteins (I0SIX2, W1TS23, and A0A3S4MEI3) were found to contain the LPXTG cell wall anchor motif, CHAP domain, and Glycosyl hydrolase family 25 (GH25), respectively. This combination of domains suggests a coordinated functional module with potential capabilities for targeted adhesion and structural modulation, implying that SA-EVs may interact with tumor cell surfaces or extracellular matrix components and locally modulate structural features. In addition, although present at lower abundance, I0SIR8 harbors the well-characterized virulence-associated domain virulence factor BrkB, indicating that these vesicles may originate from virulence-associated bacterial systems and possess potential host-interaction properties (**Figure 6D**). Collectively, these findings demonstrate that SA-EVs contain multiple proteins associated with cellular adhesion, structural modulation, and virulence-related features, supporting their potential to participate in tumor microenvironment interactions, including cytomodulatory effects and regulation of tumor cell behavior.

## 4 Discussion

This study integrated *in vitro* and *in vivo* functional assays, transcriptomics, and proteomics, to systematically elucidate the critical role and molecular mechanism of SA-EVs in GC progression. Our findings revealed that, SA functioned as a distinct pro-tumorigenic pathogen. SA-EVs were efficiently internalized by GC cells and, via their protein cargo, activate the JAK-STAT signaling pathway, thereby driving GC cell proliferation, migration, invasion, and EMT. Depleting the protein content of SA-EVs or blocking the JAK-STAT pathway effectively reverses these pro-tumorigenic effects. Collectively, our findings not only established, from an ecological niche perspective and using multi-cohort data from China, the pivotal role of Streptococcus in GC progression, but also revealed a novel mechanism whereby SA-EVs function as bacterial-host communication vectors that deliver bioactive protein components to activate the JAK-STAT axis, thereby driving malignant progression of GC.

Previous studies have confirmed that SA was significantly enriched in the gastric mucosa of patients with GC and promotes GC progression through multiple mechanisms (Fu et al., 2024; Yuan et al., 2024; Zhou et al., 2026). SA-EVs serve as critical functional vectors mediating SA-host cell interactions. Gong et al. focused on SA-EV-induced non-neoplastic gastritis and verified that virulence-associated proteins loaded within SA-EVs trigger gastric mucosal inflammatory injury and macrophage-driven inflammatory responses (Gong et al., 2026). A recent landmark study reported a potential mechanism by which SA-EVs promote GC progression and immune evasion. While the core conclusion of that study aligns with ours that SA-EVs exert important pro-tumorigenic effects in GC (Xie et al., 2025), the two studies differ substantially in mechanistic focus and research strategy. That work centered on a single effector molecule, saSNF2, and elucidated its epigenetic regulatory mechanism targeting the PD-L1 immune checkpoint. In contrast, we systematically characterized the overall effects of SA-EVs on multiple malignant phenotypes of GC cells and identified a JAK-STAT pro-tumorigenic pathway driven by the protein cargo of SA-EVs, thereby expanding the mechanistic landscape of SA-EVs action in GC. These findings collectively suggest that SA-EVs may drive gastric carcinogenesis through multiple parallel effector pathways.

In tumor malignant progression, cell proliferation, invasion, and metastasis are three indispensable biological processes that collectively determine tumor initiation and development (Li et al., 2024). Notably, postoperative tumor metastasis, recurrence, and therapy resistance are major obstacles limiting long-term survival of GC patients (Sexton et al., 2020). EMT is a key driver of tumor metastasis (Zhang et al., 2025), acting through downregulation of E-cadherin and upregulation of N-cadherin and Vimentin, leading to loss of cell adhesion and acquisition of migratory and invasive capabilities, thereby enabling cancer cells to breach the basement membrane and initiate metastasis (Mittal, 2018; Fontana et al., 2024). In contrast to direct action by live bacteria, SA-EVs, as bacteria-secreted nanoscale vesicles, deliver active molecules precisely into GC cells via membrane fusion or endocytic pathways, thereby exerting direct regulatory effects.

EVs are nanoscale carriers that transport diverse effector molecules, including DNA, RNA, proteins, and lipids (Jeong et al., 2024). These components function as an integrated unit, delivering complex bacterial instructions to host cells through synergistic interactions and exerting broad biological effects (Kulp and Kuehn, 2010; Dhital et al., 2021). To identify the key components responsible for the pro-tumorigenic effects of SA-EVs, we fractionated SA-EVs and performed functional validation. Our results demonstrated that the protein cargo serves as the critical effector mediating SA-EV-driven GC progression. Proteomic analysis combined with domain annotation revealed that SA-EV-associated proteins are enriched in multiple functional domains, including LPXTG cell wall anchor motifs and CHAP domains, which are typically involved in bacterial surface anchoring and cell wall remodeling. In addition to adhesion-related features, several SA-EV proteins harbor domains associated with pathogenic potential and interaction with host cell surfaces. Notably, the protein annotated as a ribonuclease BN-like family protein (I0SIR8) was found to contain a BrkB-like domain, implying possible functional similarity to known virulence factors involved in host cell interaction and invasion (Ni et al., 2021). Furthermore, surface antigen-like proteins enriched in SA-EVs not only enhance bacterial survival in acidic environments and *in vivo* virulence through involvement in peptidoglycan synthesis but also serve as key factors in antibiotic resistance in certain Clostridium species, thus playing multifaceted roles in bacterial pathogenicity, environmental adaptation, and drug resistance (Chia et al., 2017; Kijek et al., 2019). Collectively, these findings support a model in which SA-EVs establish contact with host cells through adhesion-associated domains, deliver effector proteins carrying virulence-associated domains and may trigger tumor-associated cellular stress responses that ultimately promote malignant transformation.

To elucidate the mechanism by which SA-EVs promote GC proliferation, invasion, and metastasis, we performed RNA-seq on tumor tissues from SA-EV-treated and control mice. Differential expression analysis followed by KEGG enrichment identified the JAK-STAT signaling pathway, which is closely associated with tumor metastasis and proliferation. Persistent activation of the JAK-STAT pathway is a key driver of malignant tumor progression (Amoyel et al., 2014; Sunny and Michelle, 2018). In various cancers, this pathway is aberrantly activated through genetic mutations, epigenetic alterations, or sustained stimulation by upstream cytokines (Chen et al., 2023), thereby remodeling the tumor microenvironment and synergistically promoting GC proliferation, invasion, and metastasis (Weilie et al., 2024). Consistently, we demonstrated that the protein cargo of SA-EVs activates the JAK-STAT signaling pathway, thereby promoting GC cell metastasis, invasion, and proliferation. Notably, treatment with AG490, a JAK-STAT pathway inhibitor, significantly reversed the pro-tumorigenic effects of the SA-EV protein cargo, confirming that the JAK-STAT pathway serves as the core downstream signaling axis mediating SA-EV-driven GC progression.

How do SA-EVs activate the JAK-STAT pathway intracellularly via their protein cargo? Given that SA-EVs are efficiently internalized by GC cells, their protein components are directly exposed to the host cytoplasmic environment. Based on our proteomic findings, we propose the following two speculative molecular hypotheses, both of which remain to be experimentally validated. *H.pylori*, a well-established carcinogenic factor in GC, has been reported to promote genomic instability through its virulence factors by inducing DNA damage and interfering with DNA repair mechanisms, while also activating multiple oncogenic signaling pathways, including JAK/STAT (Yasir et al., 2025). The nucleic acid metabolism-related proteins carried by SA-EVs, such as single-stranded DNA-binding protein, CRISPR-associated endonuclease, PriA helicase, and exonuclease, could theoretically exert analogous effects, however, this remains a speculation based solely on protein functional annotation and lacks direct experimental support. If delivered into the host cytoplasm or nucleus, these proteins might participate in the formation of aberrant nucleic acid structures or directly induce DNA damage, thereby activating the JAK-STAT pathway. This hypothesis links the protein cargo of SA-EVs to the known carcinogenic mechanisms of *H. pylori*, suggesting that different pathogenic bacteria may share partially overlapping pathogenic strategies. However, this speculation lacks direct support from cross-species comparative functional genomics and should not be overinterpreted as evidence of convergent evolution. Additionally, peptidoglycan-remodeling enzymes carried by SA-EVs, including D-alanyl-D-alanine carboxypeptidase and lysozyme, could theoretically generate immunologically active muramyl peptides upon entering the cytoplasm. These peptides serve as classic ligands for NOD1/NOD2 receptors and can cross-talk with the JAK-STAT pathway via RIP2 (Kosuke et al., 2018; Qin et al., 2019). Compared to the DNA damage pathway, this mechanism is more direct, and the role of the NOD signaling pathway in GC cells has been previously established (Peng et al., 2012; Kosuke et al., 2018; Ah-Ra et al., 2026). These proposed mechanisms may collectively mediate sustained JAK-STAT activation by SA-EVs, a hypothesis that warrants further validation.

The study also has several limitations. First, the upstream direct molecular mechanism by which SA-EVs activate the JAK-STAT signaling pathway remains to be precisely elucidated. Although we have proposed the speculative mechanistic hypotheses based on proteomics and validated the key mediating role of the JAK-STAT pathway using inhibitor experiments, the core effector proteins within SA-EVs responsible for pathway activation have not yet been identified. Future studies should employ gene knockout, site-directed mutagenesis, and surface plasmon resonance to identify the critical proteins and characterize their direct binding modes with host intracellular receptors. In addition, the hypotheses proposed in this study such as the DNA damage hypothesis should be directly validated using the comet assay. Second, more direct *in vivo* evidence is needed to support the role of SA-EVs in GC progression. While our tumor-bearing mice models have confirmed the pro-tumorigenic function of SA-EVs, the gastric microbiota constitutes a complex ecosystem with intricate synergistic or antagonistic networks among its members. SA colonizes the gastrointestinal tract, and the SA-EVs it releases directly interact with the gastrointestinal mucosa. Although we have validated the effect of SA-EVs in isolation, whether other microbial members in the *in vivo* environment synergize with or antagonize SA-EV function, as well as the actual secretion levels, diffusion range, and targeted enrichment efficiency of SA-EVs within the gastric microbial niche, remain to be validated using strategies such as *in situ* tracking. Third, the packaging mechanism of proteins into SA-EVs remains unclear that whether these proteins are passively and randomly entrapped during bacterial lysis or membrane budding, or are selectively packaged via an active sorting mechanism. However, because the present study did not perform a direct comparative quantitative analysis between the SA-EV proteome and the SA whole-cell lysate, we are currently unable to distinguish between these two possibilities, and it is one of the key priorities for future investigation.

In summary, this study systematically elucidates how SA-EV protein cargo activates the JAK-STAT signaling pathway to drive malignant progression of GC. These findings not only deepen our understanding of the role of SA-EVs in GC development and progression but also provide novel potential targets for microecological intervention and targeted therapy of GC. Nevertheless, further mechanistic dissection and *in vivo* validation are required to solidify its scientific value and clinical potential.

## 5 Conclusions

SA activates the JAK-STAT signaling axis by delivering protein components via secreted SA-EVs, thereby driving GC cell proliferation, migration, invasion, and EMT-related phenotypes.

## Data Availability

The datasets presented in this article are not readily available because they are part of an ongoing research project and are currently being used for further analyses. Requests to access the datasets should be directed to the first author, FL.
