# Supplementary material for: Streptococcus anginosus extracellular vesicles deliver protein to promote gastric cancer progression
Source: Front Cell Infect Microbiol. 2026 Sep 30;16:1917293. doi: 10.3389/fcimb.2026.1917293 (PMC13661460; doi:10.3389/fcimb.2026.1917293)
Supplement: Supplementary file 1 [file SupplementaryFile1.docx]

Supplementary Material

***Supplementary Methods***

**1.1 Data collection and microbial diversity analysis**

Public gastric mucosal microbiome datasets were retrieved from the European Nucleotide Archive (ENA) and NCBI Sequence Read Archive (SRA) under accession numbers PRJEB26931 and PRJNA634837, which represent two independent Chinese cohorts. Samples from superficial gastritis and GC were defined as the Control and Tumor groups. The datasets were generated using 16S rRNA gene sequencing targeting the V3-V4 or V4 regions. The demux summarize function in QIIME 2 (version 2025.4) was used to visualize sequencing data quality, and sequence fragments with a quality score greater than 20 were retained for subsequent DADA2 denoising. For taxonomic assignment, we used the Naive Bayes classifiers trained on full-length sequences of SILVA 138 99% OTUs, which are provided by QIIME 2 (https://library.qiime2.org/data-resources). In the following analyses, samples with a sequencing depth of fewer than 2,000 reads were removed, and the remaining samples were rarefied to a uniform depth.

Alpha diversity was calculated at the Amplicon Sequence Variants (ASV) level and beta diversity was evaluated using Bray-Curtis dissimilarity based on relative abundance. Both analyses were performed using microeco (v1.15.0). Group differences in community composition were tested using PERMANOVA with 999 permutations via the adonis2 function in vegan (v2.6-4), and dispersion homogeneity was assessed accordingly.

**1.2 Phylogenetic null model analysis of community assembly**

Microbial community assembly processes were inferred using a phylogenetic null model approach implemented in iCAMP (v1.5.12). The β-nearest taxon index (βNRI) and Raup–Crick metric based on Bray-Curtis dissimilarity (RCbray) were calculated with 1,000 randomizations. Deterministic processes were defined as |βNRI| > 2, including heterogeneous selection (βNRI > 2) and homogeneous selection (βNRI < −2). Stochastic processes were defined as |βNRI| ≤ 2. Within stochastic processes, RCbray > 0.95 indicated dispersal limitation, RCbray < −0.95 indicated homogenizing dispersal, and intermediate values indicated ecological drift.

**1.3 Differential taxa identification**

Differentially abundant taxa between Control and Tumor groups were identified at the genus level using the LEfSe implemented in microeco, with an LDA score threshold of 2. P-values were adjusted using the false discovery rate (FDR), and taxa with adjusted *P* < 0.05 were considered significant. taxa enriched in the Tumor group were retained for downstream analysis. The relative abundances of the selected pathogenic genera were extracted and visualized using ggplot2 (v3.5.2). Statistical significance was assessed using the Wilcoxon rank-sum test.

**1.4 Machine learning and model interpretation**

All genus-level annotations were extracted from the species abundance matrices of the two cohorts, and batch effects were corrected using the adjust_batch function in MMUPHin (v1.14.0), with cohort name as the batch variable, disease group as a covariate, and all other parameters kept as default. Relative abundance data were transformed by adding a pseudocount of 1 × 10⁻⁶, followed by log10 transformation and Z-score normalization. A Random Forest classifier was constructed using scikit-learn in Python. Stratified five-fold cross-validation was applied based on disease status. Hyperparameters were optimized using GridSearchCV, and model performance was evaluated using receiver operating characteristic (ROC) curves and the area under the curve (AUC). Model interpretability was assessed using SHAP (SHapley Additive exPlanations) with the TreeExplainer algorithm.

**1.5 Characterization of SA-EVs**

10 μL of the SA-EV suspension was placed on a copper grid and allowed to settle for 1 min, after which the excess liquid was absorbed with filter paper. Then, 10 μL of uranyl acetate (Cat: GZ02625-5, Zhongjingkeyi) was added onto the grid and left for 1 min to negatively stain the SA-EVs, and the excess liquid was again absorbed with filter paper. After drying at room temperature for 8 min, the samples were observed under a transmission electron microscope (HT-7700, Hitachi) operating at an accelerating voltage of 80 kV.

**1.6 Nanoparticle tracking analysis (NTA)**

The SA-EVs suspension was diluted 100-fold with sterile PBS and filtered through a 0.22μm filter. The prepared sample was injected into the sample cell of the Nanoparticle Tracking Analyzer (Particle Metrix ZetaView-PMX 130-Mono laser, Bavaria, Germany). By finely adjusting the microscope focus, camera sensitivity, and detection threshold, the particle light spots on the screen were clear and moderate in number (approximately 20-100 per frame). A PBS blank control was established to exclude background interference. Finally, a video was recorded, and the software automatically analyzed the Brownian motion of the particles to calculate the size distribution and particle concentration of the bacterial extracellular vesicles (bEVs).

**1.7 Cellular uptake of bEVs**

In the SA-EVs uptake experiment, the PKH26 dye (Cat: E0023, MilianBio) was first used to label SA-EVs. 2 μL PKH26 was mixed with 20 μL of diluent to prepare the staining working solution, which was thoroughly mixed with SA-EVs (SA-EVs concentration: 50 μg/mL) and incubated in the dark at room temperature for 10 min. Subsequently, unbound dye was removed by ultracentrifugation at 120,000 g for 90 min. Human GC cell lines AGS and MKN-45 were cultured in serum-containing medium to approximately 60% confluence, then switched to serum-free medium for overnight starvation to synchronize the cellular state. After co-incubating the labeled SA-EVs with the cells, cytoskeleton and nuclear staining were performed. The cells were washed with PBS, fixed with 4% paraformaldehyde(Cat: BL539A, biosharp), permeabilized with 0.2% Triton X-100 (Cat: T109026, Aladdin), and incubated with phalloidin (Cat: E0032, MilianBio) in the dark at 37 °C for 30 min to label F-actin. The nuclei were then stained with 1 μg/mL Hoechst 33258 (Cat: E0033, MilianBio). After mounting, the internalization of SA-EVs was observed and recorded using a laser scanning confocal microscope.

**1.8 Cell viability assay**

AGS and MKN-45 cells in the logarithmic growth phase were digested with trypsin and seeded into 96-well plates at a density of 1×10^^4^ cells per well. The cells were pre-cultured at 37 °C with 5% CO_2_ for 24 hours to ensure adhesion. Subsequently, the cells were treated with different concentrations of SA and SA-EVs according to the experimental grouping. After 24 hours of treatment, 10 µL of CCK-8 reagent (Cat: CCK-8, Dojindo) was added to each well, gently mixed, and incubated in the dark at 37 °C for 2 hours. The absorbance values were measured at 450 nm using a microplate reader.

**1.9 Wound healing assay**

The concentrations of AGS and MKN-45 cells were adjusted to 1 × 10⁵ cells/mL, and 100 μL per well was seeded into cell culture inserts within a 24-well plate. After overnight incubation, the cell culture inserts in the 24-well plate were removed using sterile forceps. The cells in the 24-well plate were washed three times with PBS, followed by the addition of serum-free medium containing SA (MOI=50) or SA-EVs (concentration: 50 μg/mL) for a 2 h co-culture. After removing the medium containing SA or SA-EVs, serum-free medium was added for continuous culture. A blank control group was established. The cells in each group were observed and photographed at 0 h and 24 h using an inverted microscope.

**1.10 Transwell invasion assay**

After co-culturing AGS and MKN-45 cells with SA (MOI=50) or SA-EVs (concentration: 50 μg/mL) for 2 h, the culture medium was removed. The cells were digested, resuspended in serum-free medium, and the cell concentration was adjusted to 5×10^5^ cells/mL. A 200 μL volume of the cell suspension was inoculated into the upper chamber of a Transwell insert (Cat: 14341, Zhenxuan) pre-coated with Matrigel and featuring an 8.0 μm pore size. A 600 μL volume of medium containing 10% FCS was added to the lower chamber, and the cells were co-cultured for 48 h. A blank control group was set up. The Transwell inserts were taken out, fixed with 4% paraformaldehyde, and then stained with 0.1% crystal violet (Cat: 548-62-9, Selleck). Finally, the cells were observed, photographed, and counted under an inverted microscope.

**1.11 EdU assay**

AGS and MKN-45 cells were seeded into 24-well plates at a density of 5 × 10³ cells per well. After overnight incubation, they were co-cultured with SA (MOI=50) or SA-EVs (concentration: 50 μg/mL) for 2 h. Then, the cells were incubated with complete medium containing 10 μM EdU for 2 h. The medium was discarded, and the cells were fixed with 4% paraformaldehyde for 20 min, followed by permeabilization with a PBS solution containing 0.3% Triton X-100 for 15 min. Next, an EdU reaction cocktail was used to incubate the cells in the dark at 37°C for 60 min to label proliferating cells. Finally, the nuclei were counterstained with DAPI, and after mounting, the cells were observed and images were captured using a fluorescence microscope.

**1.12 Establishment of animal model and experimental grouping**

After one week of adaptive feeding, 24 BALB/c nude mice (6-8 weeks old) were subcutaneously injected with 100 μL of MFC GC cells (5 × 10⁷ cells/mL) to establish the GC-bearing mouse model. Tumors formed within approximately one week. The mice were then randomly divided into three groups (n = 8 per group): control, SA-EVs, and SA. The control group received intratumoral injections of 100 μL PBS, the SA-EVs group received 100 μL SA-EVs (50 μg/mL), and the SA group received 100 μL SA suspension (1×10⁹ CFU/mL), all administered once daily. Body weight and tumor volume were measured daily until the maximum tumor volume exceeded 1500 mm³. Tumor tissues were collected for further analysis. All animal experiments were approved by the Animal Experimental Ethics Committee of Nanfang Hospital, Southern Medical University (approval Number: IACUC-LAC-20250917-006).

**1.13 Immunohistochemical staining**

The harvested tumor tissues were fixed in 4% paraformaldehyde, embedded in paraffin, sectioned, and subjected to immunohistochemical staining. Briefly, sections were dewaxed, rehydrated, and subjected to antigen retrieval, then cooled to room temperature. Endogenous peroxidase activity was blocked with 3% H₂O₂ (Cat: CS7730, G-CLONE), followed by blocking with 3% BSA for 30 min at room temperature. The sections were incubated with diluted primary antibodies against PCNA (Cat: 24036-1-AP, Proteintech) or Ki-67 (Cat: 27309-1-AP, Proteintech) overnight at 4°C in a humidified chamber. After washing with PBS, the sections were incubated with HRP-conjugated secondary antibodies (Cat: AS014, Abclonal) for 50 min at room temperature. Finally, the signals were visualized using DAB substrate, and the nuclei were counterstained with hematoxylin. The sections were then dehydrated through graded ethanol, cleared in xylene, mounted with neutral resin, and observed under a light microscope.

**1.14 Western blot**

Total proteins were extracted from mouse tumor tissues or cells using RIPA lysis buffer and quantified by the BCA method. Equal amounts of protein were separated by SDS-PAGE and transferred onto PVDF membranes. After blocking with 5% non-fat milk, the membranes were incubated overnight at 4°C with primary antibodies against N-Cadherin (Cat: 22018-1-AP, Proteintech), E-Cadherin (Cat: A3044, Abclonal), Vimentin (Cat: 10366-1-AP, Abclonal), JAK2 (Cat: AF6023, Affinity), p-JAK2 (Cat: AF3024, Affinity), STAT3 (Cat: AF6294, Affinity), and p-STAT3(Cat: AF3293, Affinity), followed by incubation with secondary antibodies for 1 h at room temperature. The protein bands were visualized using ECL chemiluminescence, and densitometric analysis was performed using ImageJ software with GAPDH (Cat: 60004-1-Ig, Proteintech) as the internal control.

**1.15 RNA-seq Analysis**

Tumor tissues from the control and SA-EV-treated groups were collected for transcriptome analysis. mRNA was enriched using Oligo dT, followed by fragmentation, cDNA synthesis, end repair, and adapter ligation. Strand‑specific libraries were constructed using the dUTP method. After quality control, sequencing was performed on an Illumina platform. Bioinformatics analysis included quality control with fastp, alignment with HISAT2, quantification with featureCounts, and differential gene expression screening using DESeq2 (|log₂FC| ≥ 1, FDR ≤ 0.05). GO and KEGG enrichment analyses were subsequently performed.

**1.16 Elimination of Proteins, RNA, DNA, or Lipids from SA‑EVs**

To specifically eliminate biomolecules from SA‑EVs, the samples were resuspended in PBS and subjected to five freeze‑thaw cycles to fully release the contents. Subsequently, proteinase K (Sigma, 0.5 mg/mL), RNase A (Takara, 10 μg/mL), or DNase (Promega, 10 units/mL) was added and incubated at the respective optimal temperatures to degrade proteins, RNA, or DNA, followed by heat inactivation or addition of inhibitors. For lipid removal, fumed silica (S5130, Sigma, 20 mg/mL) was mixed with the samples overnight, and after centrifugation, the lipid‑free supernatant was collected via sterile filtration.

**1.17 Immunofluorescence (IF)**

AGS and MKN‑45 cells were seeded into 24‑well plates at 5 × 10³ cells per well. After overnight incubation, the cells were subjected to the corresponding treatments and then processed for immunofluorescence staining. For double staining, cell slides were permeabilized at room temperature, blocked with 10% goat serum for 30 min, and then incubated overnight at 4°C in the dark with a mixture of primary antibodies against PCNA (Cat: 60097‑1‑IG, Abmart) and Ki‑67 (Cat: 60097‑1‑IG, Proteintech). After washing with PBS, the slides were incubated with a mixture of fluorophore‑labeled secondary antibodies, including goat anti‑mouse Alexa Fluor 488 (Cat: B100803, Wuhan Baiqiandu Bio) and goat anti‑rabbit Cy3 (Cat: B100802, Wuhan Baiqiandu Bio) for 50 min at room temperature in the dark. Nuclei were counterstained with DAPI. After a final wash, the slides were mounted with an anti‑fade mounting medium and observed under a fluorescence microscope for image acquisition.

**1.18 Proteomic Analysis of SA‑EV Protein Components**

Purified SA-EVs (130 μL) were lysed in urea buffer (final concentration > 6M) containing 1 mM PMSF, followed by homogenization and sonication on ice for 45 min. After centrifugation at 12,000 × g for 10 min at 4 °C, the supernatant was collected. Proteins were reduced with 10mM DTT at 56 °C for 1h and alkylated with 20mM iodoacetamide in the dark at room temperature for 40 min, with excess iodoacetamide quenched by additional 10mM DTT. Proteins were then enriched using SP3 magnetic beads and digested on-bead with a Trypsin/Lys-C mixture (2 μg) in 50 mM NH₄HCO₃ at 37 °C for 18h. The resulting peptides were acidified to 0.1% TFA, desalted using C18 Stage-Tips, and dried under vacuum at 45 °C. Protein quality was assessed by SDS-PAGE followed by Coomassie brilliant blue and silver staining.

Desalted peptides were analyzed on a Thermo Fisher Scientific Easy-nLC 1200 system coupled to a Q Exactive mass spectrometer. Peptide separation was performed using a 150 μm i.d. × 170 mm analytical column packed with Reprosil-Pur 120 C18-AQ (1.9 μm) at a flow rate of 600 nL/min. A 66-min linear gradient was employed with mobile phase A (0.1% formic acid in water) and mobile phase B (0.1% formic acid in 80% acetonitrile). Mass spectra were acquired in data-dependent acquisition mode. Full MS scans were performed at a resolution of 70,000 (AGC target 3 × 10⁶, maximum injection time 100 ms, scan range 300-1800 m/z), followed by MS/MS scans of the top 20 precursors at a resolution of 17,500 (AGC target 1 × 10⁵, maximum injection time 50 ms, normalized collision energy 28).

Raw files were processed with MaxQuant software, searching against the UniProt database (taxonomy ID 1328). Search parameters included: fixed modification of carbamidomethylation on cysteine; variable modifications of methionine oxidation and protein N-terminal acetylation; trypsin as the enzyme with a maximum of 2 missed cleavages; precursor and fragment mass tolerances of 20 ppm; and a FDR of 1% at both peptide and protein levels.

**1.19 Statistical Analysis**

All statistical analyses were performed using GraphPad Prism 10.0 or IBM SPSS Statistics 22.0. Quantitative data are presented as mean ± SD, with specific representations indicated in the corresponding figure legends. Comparisons between two groups were conducted using Student’s t-test, while comparisons among multiple groups were performed using one-way analysis of variance. Categorical variables were analyzed using the chi-square test. A threshold of *P < 0.05* was considered statistically significant.

***Supplementary Results***

**2.1 GC exhibits selection-driven alterations in microbial community structure**

To characterize microbial features associated with GC, two independent antral microbiome cohorts were analyzed in parallel (Supplementary Figure 3A). Consistent alterations in microbial composition were observed across both cohorts, with the Tumor group exhibiting marked differences compared with controls at multiple taxonomic levels (Supplementary Figure 3B). At the phylum level, Bacteroidetes and Actinobacteria were enriched; at the family level, Streptococcaceae and Prevotellaceae were increased; and at the genus level, Streptococcus, Prevotella, and Lactobacillus all showed elevated relative abundances in the Tumor group.

Alpha diversity analysis further demonstrated a consistent reduction in microbial diversity in the Tumor group across both cohorts, as indicated by decreased Chao1 richness, Pielou’s evenness, and Shannon diversity indices (Supplementary Figure 3C-3E). PERMANOVA analysis confirmed a significant shift in overall community structure between Tumor and Control groups (Supplementary Figure 3F).

Phylogenetic null model analysis revealed that microbial community assembly in both groups was predominantly governed by stochastic processes, while a measurable contribution of deterministic processes was also observed (Supplementary Figure 3G). Notably, homogeneous selection dominated the deterministic fraction, suggesting that the gastric tumor microenvironment imposes selective pressures on specific microbial taxa, thereby shaping potential disease-associated microbial signatures. These findings highlight the need to further identify key taxa driven by such selection processes.

**2.2 Streptococcus is an important microbial signature of the GC microenvironment**

Based on the consistent community-level alterations observed across the two cohorts, we next performed differential taxonomic analysis to identify candidate taxa. LEfSe analysis identified 14 genera significantly enriched in the Tumor group in the PRJEB26931 cohort, among which Streptococcus was the only genus widely reported to be associated with gastrointestinal diseases (Supplementary Figure 3I), and was therefore selected for further investigation.

Although no taxa reached statistical significance in the PRJNA634837 cohort, subsequent abundance analysis demonstrated that the trend of Streptococcus was consistent with that observed in PRJEB26931, showing a stable increase in the Tumor group across both cohorts (Supplementary Figure 3H). This cross-cohort consistency suggests that Streptococcus represents a reproducible tumor-associated microbial feature. Moreover, this observation is in line with the previously identified dominance of homogeneous selection, indicating that Streptococcus may be preferentially enriched under tumor-associated environmental pressures.

To evaluate the predictive capacity of the microbial community and to quantify the contribution of Streptococcus, we further conducted machine learning analysis. Prior to modeling, the two cohorts were integrated following batch-effect correction. The resulting models effectively discriminated Tumor from non-Tumor samples (AUC = 0.85), indicating strong predictive performance of the microbiome (Supplementary Figure 3J). SHAP analysis based on a random forest model further identified Streptococcus as a key contributing feature, with increased normalized abundance associated with a higher probability of Tumor classification (Supplementary Figure 3K).

***Supplementary Figures***

**Supplementary Figure 1**

**Supplementary Figure 2**

**Supplementary Figure 3**

***Supplementary Figure legends***

**Supplementary Figure 1** The protein cargo of SA-EVs is the key active component driving GC malignant progression in AGS cells.

(S1A-B) Wound healing assay and quantitative analysis. SA-EVs were subjected to repeated freeze-thaw cycles to disrupt vesicle structure, followed by specific removal of proteins, RNA, DNA, or lipids using proteinase, RNase, DNase, or silica, respectively, and then co-cultured with AGS cells. The protein cargo of SA-EVs significantly enhanced the migration ability of AGS cells (scale bar: 250 μm). (S1C-D) Transwell invasion assay and quantitative analysis showing that the SA-EV protein cargo significantly enhanced the invasion ability of AGS cells (scale bar: 200 μm). (S1E-F) EdU assay and quantitative analysis showing that the SA-EV protein cargo significantly enhanced the proliferative activity of AGS cells (scale bar: 100 μm). (S1G-H) Immunofluorescence and quantitative analysis of proliferation markers PCNA and Ki-67 in AGS cells treated with control, SA-EVs, or SA-EV protein cargo (scale bar: 100 μm). (S1I-K) Western blot analysis and quantitative analysis of EMT-related markers (N-cadherin, Vimentin, and E-cadherin) in AGS cells treated with control, SA-EVs, or SA-EV protein cargo. Data are presented as mean ± SD, n = 3. ns *P* > 0.05, * *P* ≤ 0.05, ** *P* ≤ 0.01, ****P* ≤ 0.001.

**Supplementary Figure 2** The protein cargo of SA-EVs drives GC malignant progression via the JAK-STAT pathway in AGS cells.

(S2A-B) Wound healing assay and quantitative analysis. The SA-EV protein cargo significantly enhanced the migration ability of AGS cells, and this effect was reversed by treatment with AG490, a JAK-STAT pathway inhibitor (scale bar: 250 μm). (S2C-D) Transwell invasion assay and quantitative analysis. The SA-EV protein cargo significantly enhanced the invasion ability of AGS cells, and this effect was reversed by AG490 treatment (scale bar: 200 μm). (S2E-F) EdU assay and quantitative analysis showing that the SA-EV protein cargo significantly increased the proliferative activity of AGS cells, and this effect was reversed by AG490 treatment (scale bar: 100 μm). (S2G-H) Immunofluorescence and quantitative analysis of proliferation markers PCNA and Ki-67 in AGS cells treated with control, SA-EV protein cargo, or SA-EV protein cargo plus AG490 (scale bar: 100 μm). (S2I-K) Western blot analysis (S2I) and quantitative analysis (S2J-K) of EMT-related markers (N-cadherin, Vimentin, and E-cadherin) in AGS cells treated with control, SA-EV protein cargo, or SA-EV protein cargo plus AG490. Data are presented as mean ± SD, n = 3. ns *P* > 0.05, * *P* ≤ 0.05, ** *P* ≤ 0.01, ****P* ≤ 0.001.

**Supplementary Figure 3** 16S rRNA sequencing and machine learning analysis reveal microbial alterations associated with GC.

(S3A) Schematic workflow of 16S rRNA sequencing analysis combined with machine learning modeling, based on datasets PRJEB26931 (n = 183) and PRJNA634837 (n = 47). (S3B) Taxonomic composition of microbiota at the phylum, family, and genus levels across different groups. (S3C-E) Alpha diversity indices including Chao1, Pielou, and Shannon. (S3F) Beta diversity analysis based on permutational multivariate analysis (PERMANOVA) demonstrating differences in microbial community structure between groups. (S3G) Sankey diagram illustrating ecological processes in microbial community assembly. (S3H) Relative abundance of Streptococcus between groups. (S3I) Linear discriminant analysis effect size (LEfSe) identifying differentially abundant taxa between groups in the PRJEB26931. (S3J) Receiver operating characteristic (ROC) curve based on the random forest model evaluating the predictive performance. (S3K) SHAP dependence scatter plot showing the contribution of key microbial features to the model output, with the left Y-axis representing SHAP values. ns P > 0.05, * P ≤ 0.05, ** P ≤ 0.01, ***P ≤ 0.001.
